# The relationship between physical function and psychological symptoms in Parkinson’s: Perceptions of People with Parkinson’s and Carers

**DOI:** 10.1371/journal.pone.0310578

**Published:** 2025-04-15

**Authors:** Philip Hodgson, Alastair Jordan, Charikleia Sinani, Divine Charura

**Affiliations:** 1 York St John University, York, United Kingdom; 2 Tees, Esk and Wear Valleys NHS Foundation Trust, West Park Hospital, Darlington, United Kingdom; Mie University Hospital: Mie Daigaku Igakubu Fuzoku Byoin, JAPAN

## Abstract

**Background:**

People with Parkinson’s (PwP) can experience both physical and psychological symptoms, and understanding the perspectives of people affected is crucial for improved management, and clinical outcomes.

**Objectives:**

This online survey aimed to gain a better understanding of the relationship between the subjectively experienced physical and psychological symptoms by PwP and their carers, while also considering the influence of personal roles and past symptom experiences.

**Methods:**

A UK-wide survey of 251 PwP and 62 carers was conducted. The survey focused on reported diagnosed and non-diagnosed psychological symptoms experienced, their onset, and the perceived impact of physical and psychological symptoms on one another. Responses were summarised using descriptive statistics.

**Results:**

A substantial proportion of respondents reported at least one diagnosed psychological condition (38.5%) or undiagnosed psychological symptoms (44.6%) such as anxiety and depression. Half of respondents reported perceiving a bi-directional interaction between physical and psychological symptoms, with this perception most reported in people with prior experience of psychological symptoms. Our sample shows that while PwP and carers have similar views on the impact of psychological symptoms, carers perceive the impact of physical symptoms to be greater than PwP.

**Conclusions:**

PwP and carers appear to perceive an interaction between physical and psychological symptoms in Parkinson’s, noting that psychological symptoms frequently precede Parkinson’s diagnosis but are often under-recognised. Improved awareness of the potential link between physical and psychological symptoms in PwP may help to improve assessment, and onward referral processes to enhance care. Further research may assist in identifying potential disease subtypes and allow the prediction of changes in physical and psychological presentation.

## 1. Introduction

In addition to common physical symptoms, Parkinson’s Disease (PD) can affect an individual’s mental wellbeing [1]. This can occur partly because of high rates of depression and anxiety, which can exacerbate motor symptoms and lead to social isolation [2–4]. People with Parkinson’s (PwP) experience higher rates of mental health issues, including depression, anxiety, schizophrenia, and psychotic symptoms, compared to the general population [1,5]. For example, whilst 17% of the general population will face anxiety and depression [6], this figure rises to 40% among PwP [7, 8]. It is believed that this increased likelihood of mental health symptoms is linked to the condition itself or the side effects of medications [9].

Despite these concerning statistics, current NICE guidelines for Parkinson’s in adults [10] do not offer specific recommendations for addressing mental health issues in PwP. Instead, they simply refer to generic guidelines for depression in adults with chronic health conditions and suggest access to allied health professionals (AHP’s) such as physiotherapists, and PD nurse specialists. This approach contrasts with guidance for other neurological conditions, including Multiple Sclerosis, which incorporates specific recommendations for regular cognitive, emotional, and mental health screenings [11, 12].

While evidence in older populations suggests a link between physical and psychological presentations [13], there is limited research confirming such a relationship in the PD population [14]. Available studies indicate that PwP perceive anxiety as a factor in amplifying their physical symptoms [15], including increased instances of freezing of gait [16]. Several studies have suggested a correlation between increased anxiety and greater severity of motor symptoms, as measured by the Unified Parkinson’s Disease Rating Scale (UPDRS) [17–20]. To our knowledge, this relationship has yet to be confirmed using more specific measures of physical function, such as balance and mobility assessments, or when considering other psychological symptoms associated with PD [21, 22]. Confirming this relationship using alternative measures that are widely used within clinical practice by healthcare professionals is a vital step to inform clinical practices, particularly given the aforementioned NICE guidelines. Based on the high proportion of individuals with PD affected by psychological symptoms, further research in this area could significantly enhance our understanding of the potential interaction between physical function and psychological symptoms, potentially leading to improved care strategies.

Prior to completing this work, our team conducted a systematic review [14] which highlighted that despite many studies routinely collecting data for both physical and psychological outcomes, only one study examined the relationship between these outcomes [23]. Furthermore, our exploratory meta-regression analysis of extracted baseline group-level mean data from previous studies suggested a trend for the physical ability of PwP to reduce as symptoms of depression increase.

In the study that examined the relationship between physical and psychological outcomes [23], bivariate correlation analysis for Hospital Anxiety and Depression Scale (HADS) score against performance on the Dynamic Gait Index (DGI) outcome measure were carried out. Interestingly, no significant correlation was found between DGI performance and either overall HADS score (r=0.269), HADS-A (r=0.132), or HADS-D (r=0.239) whereas significant correlations were found between self-reported motor disability (International Parkinson and Movement Disorder Society - Unified Parkinson’s Disease Rating Scale (MDS-UPDRS) Part 2) and HADS score (r=0.624), HADS-A (r=0.536), and HADS-D (r=0.481). The lack of significant correlation between the DGI and HADS outcomes suggests that clinically led assessments of physical function may not capture patients’ subjective experiences of psychological distress. In contrast, significant correlations between self-reported motor disability (measured by the MDS-UPDRS Part 2) and HADS scores indicate that those who perceive greater motor disability also report higher anxiety and depression levels. This discrepancy points to a potential mismatch between how patients view their condition and how it is captured by clinically led assessments, emphasising the need for further research to explore these differences. Understanding this relationship is crucial for improving clinical practices, as it may lead to more effective management strategies that consider both physical and psychological symptoms in PD.

Despite some research offering insights into the physical and psychological interactions experienced by PwP via interviews [15,24], to-date, no survey research has explored the perspectives of PwP and carers of PwP regarding their perceived interaction between physical and psychological symptoms in PD. A quote from interviews conducted by Sundström et al. [24] states that: “*Yeah I, I’ve always said it’s a vicious circle, the anxiety will feed the Parkinson’s and the Parkinson’s because of the symptoms will make you anxious so it’s just, vicious circle.”* This represents a significant gap in our understanding of the lived experiences of those affected by PD. Current evidence lacks detailed input from PwP and carers on their perceptions of symptom interactions, offers limited understanding of how these interactions are experienced in daily life, and provides insufficient data on how perspectives might differ between PwP and carers of PwP.

Given these under-researched areas, our study aimed to provide a platform for PwP and carers of PwP to share their experiences and insights. Through an online survey, we collected views on potential interactions between physical and psychological symptoms, as well as information on how these interactions are experienced. Understanding these factors is crucial for informing future research directions and improving clinical service delivery in PD care. Opportunities for future research include longitudinal studies that track the progression of both physical and psychological symptoms in PwP over time, and comparative studies between PwP and other neurological conditions, such as Multiple Sclerosis or Alzheimer’s disease, to provide insights into the unique aspects of symptom interactions across conditions.

The rationale for our research stems from the substantial impact that both physical and psychological symptoms have on PwP, yet the interaction between these symptoms remains underexplored. Our exploratory study aims to investigate whether PwP and their carers perceive a connection between physical and psychological symptoms, considering the influence of personal roles and past experiences with these symptoms. By conducting a UK-wide survey, we seek to gather insights directly from those affected, thereby addressing a critical gap in existing literature. This online survey aimed to gain a better understanding of the relationship between the subjectively experienced physical and psychological symptoms by PwP and their carers. Our objectives were to investigate whether individuals perceive a connection between physical and psychological symptoms, while also considering the influence of personal roles and past symptom experiences. Our hypothesis posits that PwP and their carers will report a bi-directional interaction between physical and psychological symptoms, with psychological symptoms often preceding or exacerbating physical challenges.

## 2. Materials and methods

### 2.1. Ethics

Ethical approval was given by the School of Science, Technology and Health Research Ethics Committee at York St John University (STHEC0067). Following provision of the participant information sheet, informed consent was obtained. This was completed electronically at the start of the survey with participants confirming “I meet the eligibility criteria and consent to completing the survey”. We confirm that we have read the Journal’s position on issues involved in ethical publication and affirm that this work is consistent with those guidelines.

### 2.2. Developing the Survey

To ensure that the survey considered priority areas, feedback on questions and study documentation was sought. A draft survey was produced, and feedback provided by three volunteers recruited through Parkinson’s UK from which one was a carer and two PwP. Questions posed to volunteers are available within supplementary study materials. Volunteers reported that the research purpose was clear and that they supported the completion of research in this area. Language used within the survey and associated documentation was edited based on feedback received. Changes included altering the estimated timescales for completion from 20-minutes to 30-minutes to reflect more accurately the time taken for PwP to complete the survey, ensuring consistency of question wording and suitability for a lay-audience, and removal of questions deemed to be too closely related. Following feedback, the topic areas addressed included: Demographics, Physical activity, Mental health, Symptom interactions, and Treatments. A copy of the final survey questions and flow is available in the supplementary material.

### 2.3. Recruitment and Procedures

The survey was open 24/11/2022-21/03/2024 via Qualtrics, an online survey and questionnaire tool. The survey was promoted via the Parkinson’s UK Research Support Network and associated Parkinson’s research interest groups who circulated the opportunity to participate in this study to their members via email. The Research Support Network has approximately 7,000 members (May 2022), the vast majority of whom are PwP and partners, family members and carers of those with the condition living in the UK. An email was sent to members of the research support network via a monthly e-newsletter, as well as being promoted via the Parkinson’s UK ‘Take Part Hub’.

The survey gathered responses from PwP, carers who were identified by response to the first survey question and required to confirm their eligibility. Carers were encouraged to answer each question based on their own perception about the person they provided care for.

Whilst some demographic information was collected, the survey did not collect any personal identifiable information. Participants were able to skip any questions they did not wish to answer. A copy of the survey questions and flow is available in the supplementary material.

### 2.4. Data analysis

Given the exploratory nature of this research, descriptive statistics were used to characterise the sample and data were analysed using Microsoft Excel. Unfinished surveys (where participants did not reach the final question) were excluded from the analysis. Missing data for individual questions, where participants elected not to respond but continued with the survey, were included to avoid deletion of responses to other questions. Where possible, some comparisons have been made between PwP and carers however it should be noted that this is purely via descriptive statistics, and we have not completed any inferential statistics. This analysis is crucial in establishing baseline data that can inform future research directions or interventions.

## 3 .Results

A total of 313 responses were received, of which 251 were from PwP and 62 from carers. Responses to questions are detailed in the text and table/figures below. Please note that in some instances participants were able to select more than one response per question. Of the 353 individuals who confirmed their eligibility and consented to participating, 40 surveys were incomplete and were not included within our analysis. The 88.7% completion rate indicates that the survey was engaging, easy to understand, and well-structured with a manageable length.

### 3.1. Participant demographics

Table 1 shows details of participant demographics. Of the 313 respondents, 251 were from PwP, with the remainder from carers. PwP completing the survey tended to be aged 60 or over (74.1%), whilst carers were generally younger. Overall, 57.5% of respondents were female. For carer respondents, 90.3% were female, whilst this was 49.4% in responses from PwP. Responses were overwhelmingly from those identifying as white British (93.6%), with this being evident in both groups of responders (PwP: 93.2%, carers: 95.2%). In PwP, time since PD diagnosis ranged from 0.01 years to 22.52 years, with a mean of 5.32 years. Time since PD diagnosis tended to be higher in responses from carers, with a mean of 10.56 years, potentially indicating higher levels of disability and more active carer input.

**Table 1:** Participant demographics and physical activity level/importance.

Demographic Item	Group			
	PwD		Carer	
	n	%	n	%
Total participants	251	*80.4%*	62	*19.8%*
Age				
18–29	0	*0.0%*	4	*6.5%*
30–39	2	*0.8%*	5	*8.1%*
40–49	15	*6.0%*	7	*11.3%*
50–59	48	*19.1%*	11	*17.7%*
60–69	100	*39.8%*	19	*30.6%*
70–79	71	*28.3%*	16	*25.8%*
80–89	13	*5.2%*	0	*0.00%*
90 or older	1	*0.4%*	0	*0.00%*
Prefer not to say	1	*0.4%*	0	*0.00%*
Gender				
Male	123	*49.0%*	6	*9.7%*
Female	124	*49.4%*	56	*90.3%*
Non-binary	0	*0.0%*	0	*0.0%*
Prefer not to say	1	*0.4%*	0	*0.0%*
Other	3	*1.2%*	0	*0.0%*
Ethnicity				
White: English/Welsh/Scottish/Northern Irish/British	234	*93.2%*	59	*95.2%*
White: Irish	4	*1.6%*	1	*1.6%*
White: Any other White background	6	*2.4%*	1	*1.6%*
Asian or Asian British: Indian	2	*0.8%*	0	*0.0%*
Black or Black British: Caribbean	0	*0.0%*	1	*1.6%*
Prefer not to say	5	*2.0%*	0	*0.0%*
Other	0	*0.0%*	0	*0.0%*
Reported Time (years) Since Diagnosis				
Mean (SD)	5.32 (4.36)		10.56 (9.16)	
Range	0.01-22.52		0.13-42.91	
Physical Activity Level				
Very Active	67	*26.7%*	5	*8.2%*
Quite Active	75	*29.9%*	6	*9.8%*
Average	73	*29.1%*	14	*23.0%*
Low	33	*13.1%*	21	*34.4%*
Very Low	1	*0.4%*	15	*24.6%*
Perceived Importance of Exercise				
Not important at all	1	*0.4%*	0	*0.0%*
Slightly important	1	*0.4%*	4	*6.5%*
Moderately important	16	*6.4%*	5	*8.1%*
Very important	113	*45.0%*	25	*40.3%*
Extremely important	120	*47.8%*	28	*45.2%*

### 3.2. Perceived symptom interactions

**Fig 1:**
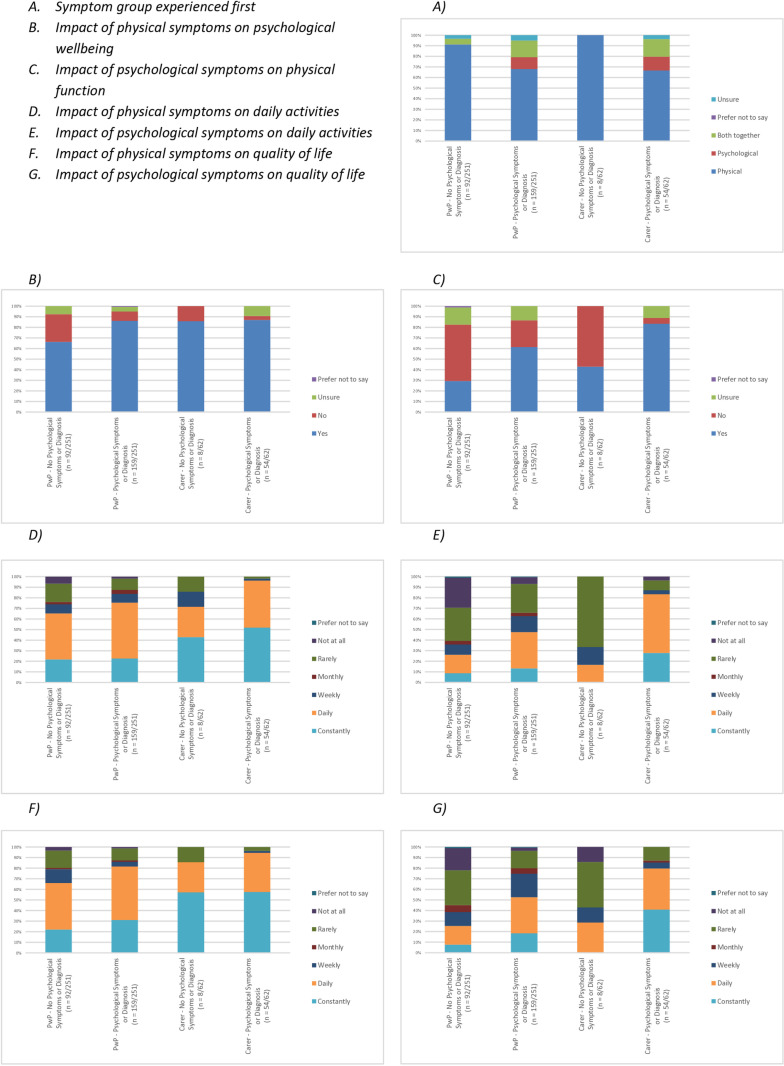
Perceived Symptom Interactions.

Fig 1 shows details of reported symptom interactions as perceived by respondents. This data provides additional subgroup analysis for individuals reporting the presence of psychological symptoms and/or diagnosis in comparison to individuals not reporting these.

#### 3.2.1. Symptoms Experienced First.

Graph A shows that despite the high prevalence of psychological diagnoses and symptoms reported pre-PD diagnosis, the majority of PwP and carers report experiencing physical symptoms of PD prior to any psychological symptoms (76.5% and 70.5% respectively). This is higher in PwP and carers not reporting psychological symptoms (91.3% and 87.5%), dropping to 67.9% and 66.7% when considering only those who reported the presence of psychological symptoms and/or diagnosis.

#### 3.2.2. Psychical and psychological symptom interaction.

Overall, both PwP and carer groups report perceiving physical symptoms to impact on psychological wellbeing (PwP: 78.9%, carers: 86.9%). This was again higher when considering PwP and carers who reported the presence of psychological symptoms and/or diagnosis (86.2% and 87.0% respectively) in comparison to those not reporting psychological symptoms and/or diagnosis (66.3% and 75.0%). When considering the inverse relationship, 49.4% of PwP and 78.7% of carers reported that they perceived psychological symptoms to impact upon physical function. Once again, this was higher in those reporting psychological symptoms/diagnosis (PwP: 61.0%, carers: 83.3%) when compared to PwP and carers not reporting psychological symptoms and/or diagnosis (29.3% and 37.5%). These results are shown in graphs B and C. Overall, 156 individuals (49.8%) reported perceiving that both physical and psychological symptoms impacted on one another. Of these, 127 (81.4%) were individuals with prior experience of psychological symptoms and/or diagnosis.

#### 3.2.3. Psychical and psychological symptom interaction (Daily Activities and Quality of Life).

Follow-up questions were asked to ascertain the extent of any impact, with questions addressing the impact of both physical and psychological symptoms on daily activities (graphs D and E) and overall quality of life (graphs F and G). Responses indicate that both groups of PwP and carers perceive a ‘constant’ or ‘daily’ impact of physical symptoms on both daily activities (PwP: 71.7%, carers: 91.9%) and quality of life (PwP: 75.3%%, carers: 91.9%). Based on responses received, a lower impact is perceived on daily activities (PwP: 39.44%, carers: 74.2%) and quality of life (PwP: 42.2%%, carers: 72.6%) as a result of psychological symptoms. These views regarding the impact of psychological symptoms again appear impacted by the presence of reported psychological symptoms/diagnosis, with individuals reporting psychological symptoms/diagnosis perceiving a greater impact of psychological symptoms on daily activities (PwP: 47.2%, carers: 83.4%) and quality of life (PwP: 52.2%, carers: 79.6%) when compared to PwP and carers not reporting psychological symptoms/diagnosis (Daily activities: 26.1% and 12.5% and quality of life 25.0% and 25.0%).

### 3.3. Psychological symptoms and diagnoses

#### 3.3.1. Presence of reported psychological diagnoses/symptoms, and frequency by condition.

Participants were asked to recall the presence of psychological diagnoses and symptoms without diagnosis overall, before being asked to select the timepoint at which these diagnoses or symptoms were given/began. These timepoints included pre-Parkinson’s diagnosis, diagnosis to 6-months post-diagnosis, 6-months to 2-years post-diagnosis, 2–5 years post-diagnosis, and over 10-years post-diagnosis. These timepoints were chosen in order to capture information relating to significant events such as immediately following Parkinson’s diagnosis, but also capture changes potentially relating to disease progression and the impact of elements such as medication. Data analysis for the presence of symptoms/diagnosis was completed by responder group, whereas timepoint data was combined responses from PwP and carers to achieve a more balanced spread across the length of time since Parkinson’s diagnosis.

**Fig 2:**
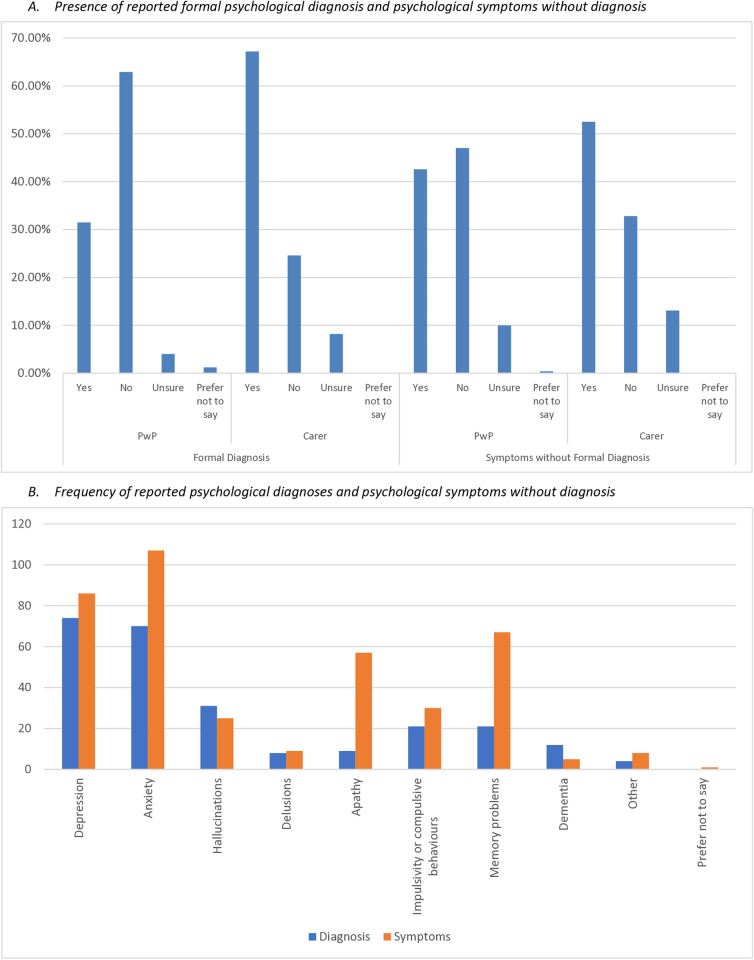
Details of psychological diagnoses and symptoms reported, and the frequency of reported psychological diagnoses and symptoms.

Fig 2 details reported psychological diagnoses received, and symptoms reportedly experienced without receiving a diagnosis. In the PwP group, 79 (31.5%) reported having received a diagnosis of psychological condition(s). Of these, the most commonly reported were anxiety (19.1%) and depression (20.3%). A total of 41 carers (66.1%) reported that the individual they provide care for had received a diagnosis of psychological condition(s). Of the diagnoses reported by carers, the most common were also anxiety (35.4%) and depression (37.1%).

A greater proportion of PwP (107, 42.6%) reported having experienced symptoms of psychological condition(s) without receiving a diagnosis. The most commonly reported symptoms in this instance were anxiety (35.9%), depression (27.1%), apathy (17.1%) and memory problems (19.1%). A total of 32 carers (52.5%) reported that the individual they provided care for had experienced symptoms of psychological conditions without receiving a diagnosis. The most commonly reported symptoms in this instance were also anxiety (27.9%), depression (29.5%), apathy (23.0%) and memory problems (31.1%). Psychological symptoms and diagnoses reported as ‘other’ included: ‘Post-Traumatic Stress Disorder’, ‘Bipolar Disorder’, and ‘Stress’, however accounted for 1.86% of responses to this question and have therefore not been considered further within data analysis.

#### 3.3.2. Timepoint of reported diagnoses/symptoms.

**Fig 3:**
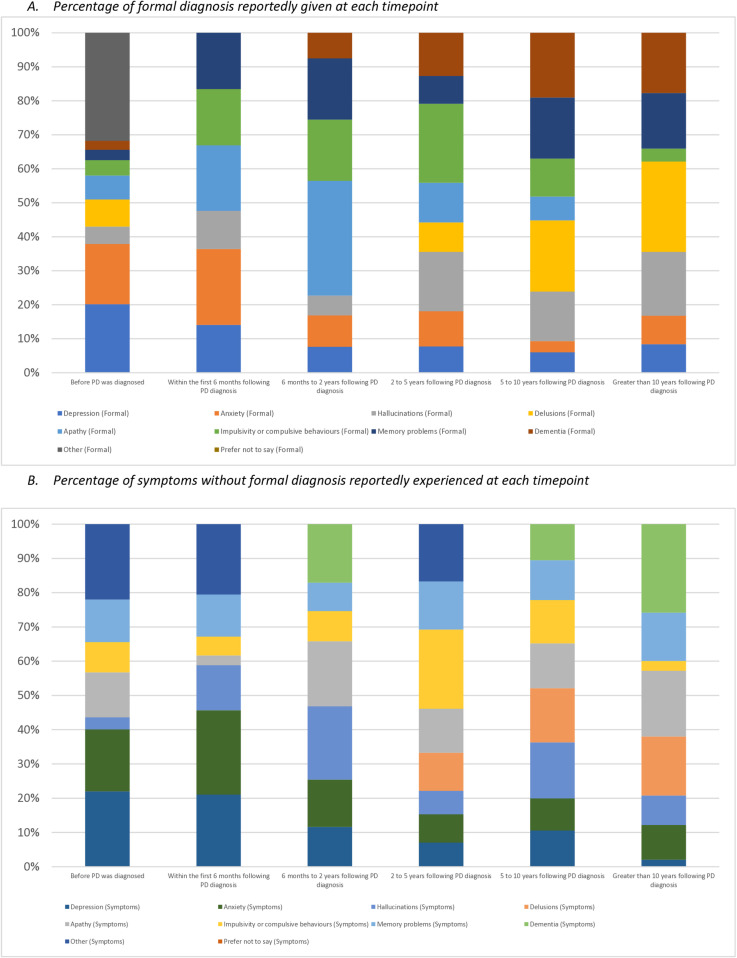
Diagnoses/Symptoms reportedly experienced at each timepoint.

Fig 3 expands on the reported diagnoses and symptoms to provide details around the timepoint at which the various diagnoses and symptoms were first given or experienced. This data takes into account the number of individuals reaching each timepoint based on the reported date of Parkinson’s diagnosis. From all responses received, the greatest likelihood of receiving a diagnosis of the following conditions was as follows: Depression – pre-PD diagnosis (63.5%), Anxiety – pre-PD diagnosis (55.7%), Hallucinations – Greater than 10y post-PD diagnosis (35.3%), and Impulsivity or Compulsive behaviours – 2-5y post-PD diagnosis (33.3%).

From all responses received across both groups, the greatest likelihood of experiencing symptoms yet not receiving a diagnosis was as follows: Depression – pre-PD diagnosis (50.0%), Anxiety – pre-PD diagnosis (41.1%), Apathy – Greater than 10y post-PD diagnosis (55.6%), and Memory Problems – Greater than 10y post-PD diagnosis (40.9%). From our data, there appears to be greater variation in the reported timeframe of symptoms experienced without diagnosis in comparison to the timepoints reported for when diagnoses were received.

### 3.4. Physical activity

Table 1 also shows details of the reported physical activity levels, alongside details of the perceived importance of physical activity. Most of the responses from PwP indicate physical activity levels of average or higher (85.7%). Although carer responses do not necessarily relate to the same individuals, responses show a possible difference between the perception of the two groups of responders, with only 41.0% of carers reporting that the individual they care has activity levels of average or higher. Despite this mismatch in reported activity levels, both groups report physical activity to be either very important or extremally important (PwP: 92.8%, carers: 85.2%).

## 4. Discussion

This online survey aimed to gain a better understanding of the relationship between the subjectively experienced physical and psychological symptoms by PwP and their carers, while also considering the influence of personal roles and past symptom experiences. To our knowledge, this is the first study to explore the perspectives of PwP and their carers, which provides a direct account of how these issues are viewed by the recipients of clinical care.

This exploratory research has improved our understanding of how PwP and carers perceive the relationship between physical and psychological symptoms experienced. From our survey results, PwP and carers appear to appreciate the link between physical function and non-motor symptoms in Parkinson’s, however both respondents reported an under-recognition of psychological symptoms experienced. The findings of this study highlight the complex interplay between physical and psychological symptoms in PD, emphasising the importance of a holistic approach to patient care. For these reasons, efforts should be made to improve not only formal symptom recognition but also to optimise signposting to appropriate services.

### 4.1. Perceived symptom interactions

#### 4.1.1. Symptoms experienced first.

There is evidence to suggest that non-motor symptoms may precede diagnosis of PD, and even the motor symptoms themselves [25]. Even though many respondents reported diagnosed or non-diagnosed non-motor symptoms prior to the diagnosis of PD, it was the motor symptoms that were reported by over 75% to be experienced first. These findings may be due to: 1) a lengthy process of diagnosing PD, where both types of symptoms are experienced before a diagnosis is made; 2) a recall bias towards motor symptoms experienced; or 3) a general lack of recognition that non-motor symptoms may be associated with PD even amongst individuals directly impacted by the condition.

#### 4.1.2. Physical and psychological symptom interaction.

Individuals who reported psychological symptoms (with or without these being diagnosed) demonstrated an understanding that such symptoms may emerge prior to any physical symptoms. Interestingly, of almost 50% of the respondents perceiving physical and psychological symptoms to impact on one another 81.4% of these respondents had prior experience of psychological symptoms. This may suggest an under-recognition of potential symptom interactions from both groups of respondents until psychological symptoms are directly experienced. If true, this potentially highlights a gap in education from clinicians when preparing people for what to expect as their condition progresses [26, 27]. Any educational practices may be relevant at all stages of the condition, including when explaining to individuals that the psychological symptoms they have experienced prior to PD diagnosis may be related to the presence of PD itself.

#### 4.1.3. Physical and psychological symptom interaction (Daily Activities and Quality of Life).

Respondents with experience of psychological symptoms perceived these to have a high impact on physical function, daily activities, and quality of life. In comparison, individuals without experience of psychological symptoms were less likely to report a perceived impact in these areas. In addition to the impact of personal experiences, there may also be an impact of an individual’s role, with PwP and carers responding differently to questions concerning the influence of physical symptoms. More specifically, responses from carers showed a greater perceived impact of physical symptoms on daily activities and quality of life in comparison to PwP. This may be a result of the carer burden experienced, or a reflection of PwP becoming more accustomed to symptoms and adjusting their lifestyles accordingly. These findings align with work by Sundström & Jola [24], who found that whilst carers and their Parkinson’s partners share some experiences, the topics differ overall. This may help to explain some of the potential difference in how the impact of symptoms may be perceived by different groups, with carers potentially more likely to view the burden of physical disability as a priority for them.

### 4.2 Psychological symptoms and diagnoses

#### 4.2.1. Presence of reported psychological diagnoses/symptoms, and frequency by condition.

It is worth noting that for some individuals, the reported symptoms experienced may have not been diagnosed. Nevertheless, the rates of anxiety and depression reported by our PwP respondents are consistent with published prevalence rates [8]. Interestingly, the majority of our carer responders reported higher rates of psychological symptoms (diagnosed and non-diagnosed) than our PwP respondents. Such findings may be because fewer carer respondents completed the survey in comparison to our PwP respondents. In addition to this, those who completed it were predominantly carers for individuals with PwP who had been diagnosed with PD for longer, and therefore more likely to have experienced more severe symptoms.

#### 4.2.2. Timepoint of reported diagnoses/symptoms.

Anxiety and depression were the most likely psychological symptoms to be reported by our PwP and carer respondents prior to a diagnosis of Parkinson’s. Evidence from studies examining the prevalence of psychological symptoms found these conditions to be frequently apparent before a PD diagnosis, and in some cases as early as 7 years prior to a PD diagnosis[25,28]. It is unclear whether anxiety and depression before a Parkinson’s diagnosis indicate the disease itself; however, their higher prevalence in people with Parkinson’s compared to the general population [29] suggests a promising area for future research. If the onset of psychological symptoms is linked to the loss of dopamine-producing cells in the substantia nigra compacta, leveraging this connection could aid the early diagnosis and management of PD before 60–80% of these cells are lost and physical symptoms emerge [30].

Evidence has shown that diagnoses of anxiety and/or depression can occur throughout the PD timeline, such as prior to PD diagnosis (prodromal phase) [28,31,32], around the time of PD diagnosis [33,34] and as the condition progresses [35]. Despite this, there is a lack of evidence regarding how these symptoms of anxiety and depression may relate to changes in physical function and vice-versa, with this relationship not being well understood. It is possible that one or a combination of reasons may explain any psycho-physical interactions throughout the PD timeline: 1) The neurodegenerative processes underlying PD contributes to the development of anxiety and depression; 2) These psychological conditions exacerbate the physical symptoms of PD; 3) The stress and uncertainty associated with the early, undiagnosed stages of PD may play a role in the emergence of these psychological symptoms; 4) Side effects of medication. Given the lack of research in this area we are currently unable to provide certainty regarding the specific interactions in-play. Future research should aim to disentangle these complex interactions to better understand the relationship between physical and psychological symptoms in PD, and to develop more effective strategies for early diagnosis and management.

Apathy and impulsivity were frequently reported in our sample, often without formal recognition. Apathy symptoms were consistent throughout the disease’s progression, while impulsivity peaked 2–5 years after diagnosis, aligning with previous research [36,37]. This pattern may reflect the effects of dopaminergic medication [38]. Hallucinations were most commonly reported 2–5 years post-diagnosis, with diagnoses peaking over 10 years later, likely due to levodopa side effects and limited treatment options that avoid additional side effects [39].

These findings highlight the importance of education and early screening of psychological symptoms so that referral to appropriate services can take place. While this research does not provide a comprehensive resource for Parkinson’s symptoms at every stage, it offers insights into self-reported symptoms often overlooked by clinical services. Additionally, we lay the groundwork for further investigation into symptom presence and clinical actions taken upon identification. Byeon [40] developed a machine learning model to predict depression risk based on motor signs, REM sleep behaviour disorders, and neuropsychological tests. As suggested by others, it may eventually be feasible to predict PD development by identifying non-motor symptoms before observable motor symptoms appear [41–44].

### 4.3. Physical activity

Previous research indicates that PwP view exercise to be vital in maintaining their physical function [45], a perspective echoed in our study by both PwP and carers. Interestingly, our study revealed differences between PwP and carers regarding reported levels of physical activity, with carers reporting lower levels of physical activity. It is important to note that the PwP and carer samples were not matched and do not necessarily reflect different perspectives of the same individuals. This discrepancy may therefore be due to our carers mainly supporting individuals with more significant physical or psychological challenges, as evidenced by the given average time since diagnosis (carers: 10.56 v’s PwP: 5.32 years). A greater time since diagnosis suggests greater physical decline [46] but does not necessarily indicate an increased severity of symptoms of anxiety and depression [47].

This work will help to provide a basis for service development alongside aiding the design of future clinical research projects ensuring that both are tailored to meet their needs. Longer-term, this work may help to provide a platform for improving diagnostic processes or identifying PD subtypes that may explain differences in the underlying cause of specific symptoms.

### 4.4. Strengths and limitations of this study

While our survey gathered a significant number of responses, indicating a strong interest in the topic, the purposive and snowball sampling methods may have attracted respondents with experience of psychological symptoms. To help alleviate concerns regarding our sample, we compared our findings with existing research to assess representativeness; for instance, participants demonstrated a strong belief in the importance of physical activity and reported rates of anxiety and depression aligned with established prevalence in PD [7, 8].

We acknowledge that our study lacks detailed statistical analysis of group responses. Given the exploratory nature of this research and the unmatched PwP/carer respondents, such analysis could yield misleading conclusions. All respondents were from the UK, which may affect the accessibility of clinical services. Nonetheless, our survey effectively met its exploratory aim by allowing PwP and their carers to share experiences regarding interactions between physical and psychological symptoms.

Due to the online survey format, we cannot confirm whether carers completed responses on behalf of PwP. We aimed to mitigate this by allowing carers to provide their own perspectives, facilitating exploratory group comparisons. This limitation highlights the need for further validation through methods like in-person qualitative interviews.

We did not collect information on the physical symptoms, or their severity experienced by PwP in our survey. Consequently, we cannot infer interactions between specific physical and psychological symptoms; however, this presents an opportunity for future research now that a general perspective has been established. We addressed potential recall bias by instructing carers to report based on direct observations rather than assumptions. The demographic skew of our participants may limit the generalisability of our findings, underscoring the need for more diverse research to better understand experiences across the Parkinson’s community.

A major strength of this study was involving PwP and carers in designing the survey, ensuring it focused on areas that mattered most to them.

## 5. Conclusions

Our survey results indicate that both PwP and carers for PwP recognise the link between physical function and non-motor symptoms, yet they report an under-recognition of psychological symptoms. Anxiety and depression were commonly experienced and diagnosed, but there was considerable variation in the timing of these diagnoses. Many respondents reported that psychological symptoms preceded their Parkinson’s diagnosis. The perceived interactions between these symptoms and their broader impacts appear to differ based on whether the respondent is a PwP or a carer, as well as their personal experience with psychological symptoms.

These findings underscore the complex interplay between physical and psychological symptoms in PD, highlighting the need for a holistic approach to patient care. The perceived bi-directional relationship between these symptom types, especially among those with prior experiences of psychological symptoms, emphasises the necessity for comprehensive assessment and management strategies. The differing perceptions of symptom impact between PwP and carers further illustrate the importance of considering multiple perspectives in care planning. By recognising the interconnectedness of these symptoms, healthcare providers can create more effective, personalised treatment plans that address various aspects of the disease, potentially enhancing the quality of life for individuals living with Parkinson’s.

Given the recognised interaction between physical and psychological symptoms, efforts should focus on improving the recognition of psychological issues and optimising referrals to appropriate services. Enhancing the assessment of psychological symptoms is essential for providing comprehensive care for PwP. Further research is needed to identify potential subtypes of Parkinson’s disease and to objectively predict how changes in psychological symptoms relate to declines in physical function, and vice versa.

Our study highlights the critical relationship between psychological symptoms and Parkinson’s diagnosis, including cases where motor symptoms may not yet be apparent. We clarify that psychological symptoms like anxiety and depression can manifest before a Parkinson’s diagnosis may be linked to the disease itself. This connection necessitates comprehensive evaluations in clinical settings, integrating mental health assessments into routine care for individuals with Parkinson’s, regardless of motor symptom presentation. Such integration may help healthcare providers better recognise and address the psychological dimensions of Parkinson’s, leading to improved outcomes and more tailored treatment strategies. Future research should continue to explore these relationships, focusing on how psychological symptoms influence both disease progression and overall quality of life for PwP.

## Supporting information

S1 File Survey Questions and Flow. (DOCX)
